# Tobacco control policy and regulation from diverse perspectives and contexts

**DOI:** 10.1136/tc-2024-058662

**Published:** 2024-03-19

**Authors:** Marita Hefler

**Affiliations:** Menzies School of Health Research, Charles Darwin University, Casuarina, Northern Territory, Australia

**Keywords:** addiction, advertising and promotion, advocacy

This issue brings together studies providing different perspectives of tobacco regulation and policy for cigarettes, heated tobacco products (HTPs), waterpipe tobacco, e-cigarettes and other products.

Taxation is cost-effective for governments while reducing smoking prevalence. Ngo *et al* provide evidence for the underused potential of this policy lever.^1^ Using the Tobacconomics scorecard and drawing on 2014-2020 data, they show that modest improvement in tax policy scores globally reduced cigarette consumption by 3.27%, but this could have been reduced by 20.74% with optimal tax policies. Low-income and middle-income countries stood to gain even more, with the potential to reduce consumption by 28.05%. In contrast to cigarettes, waterpipe tobacco taxes are under-researched. Jawad *et al* address this by modelling waterpipe taxes for Jordan, Lebanon and Palestine.^2^ They highlight the need for context specific modelling, noting that waterpipe smoking in cafes has a high industry mark-up which reduces the tax burden and therefore its potential effectiveness to reduce consumption. Consistent with Ngo *et al*,^1^ they show that waterpipe tobacco excise tax increases could raise significant government revenue while averting large numbers of premature deaths.^2^

Two papers examine tobacco pricing and smoking prevalence. In eight sub-Saharan countries, Filby analyses the relationship between smoking and tobacco prices using 2012-2018 Global Adult Tobacco Survey (GATS) data.^3^ Filby found that cigarette price is the only statistically significant policy predictor of smoking prevalence and intensity—making tax increases an attractive option in the region. In Vietnam, where male smoking prevalence is 39%, Nguyen *et al* 4 also use GATS data to explore the effect of cigarette prices on smoking uptake and cessation. Consistent with previous studies, they find that increased cigarette prices reduce smoking uptake by young people but not cessation among people who smoke. They note that the tax share of the retail price of tobacco in Vietnam is approximately one-third—less than half that recommended by WHO - providing ample room for Vietnam to increase taxes, while also strengthening and better enforcing other comprehensive tobacco control policies such as smoke-free areas and advertising bans.

On the topic of comprehensive tobacco control policy, Mengesha *et al’s* study suppports the importance of enforcement; despite a strong smoke-free law on paper, they found very high levels of non-compliance across four Ethiopian regions.^5^ The need for comprehensive advertising bans is demonstrated by Khayat *et al*.^6^ Their content analysis shows the ways in which Philip Morris International’s cigarette and IQOS advertisements evolved through four regulatory periods where IQOS went from classification as a consumer product with no advertising restrictions, to a tobacco product subject to the same regulations and advertising restrictions as cigarettes. With print media excluded from the advertising ban, QR codes were used, highlighting a regulatory gap and the need for policies to limit tobacco companies’ ability to circumvent restrictions. They also show how advertising spends shifted and targeted specific population groups,^7^ and that messaging shifted after the US Food and Drug Administration’s (FDA) modified risk tobacco product authorisation, demonstrating the influence of regulatory agencies internationally.^6^

The impact of the US FDA’s authorisation for IQOS to use marketing claims of ‘reduced exposure’ on consumer perceptions is examined by Berg *et al*.^8^ They found that reduced exposure messages resulted in lower perceived relative harm, providing evidence of the need to monitor advertising content which harnesses FDA language. Similarly, Wu *et al* 9 highlighted that FDA-reduced exposure authorisation was used to argue against a total ban on HTPs in Hong Kong, which was originally proposed in January 2018 but only passed into law in October 2021. Despite HTPs never having been formally marketed in Hong Kong, their use increased from 8.9% in 2017 to 25.3% in 2019–2020. Wu *et al* found that while a majority of respondents supported the proposed regulations, perceptions of reduced harm from HTPs were associated with lower support.^9^ The potential for US FDA decisions to be misused internationally is highlighted by Glantz and Lempert, who provide a detailed critique of the US FDA’s premarket tobacco product application process for Vuse Solo e-cigarettes.^10^ Lindblom’s accompanying commentary notes that similar critiques for FDA decision-making are now more difficult due to FDA only releasing summaries of decisions, and also notes its inadequate interpretation of protection of public health.^11^ Meshnick *et al* further extend this analysis, offering a framework for national regulators to learn from the US experience.^12^

Four papers examine policies related to menthol. Gendall and Hoek^13^ analysed tobacco company returns to the New Zealand Ministry of Health over 11 years and found that they hold a modest but significant share of tobacco sales in the country. Yang *et al* 14 surveyed people who currently smoke menthol or flavoured tobacco products and/or use non-tobacco flavoured e-cigarettes about how they would respond to three different scenarios of menthol and other flavoured tobacco product bans, with or without including flavoured and menthol e-cigarettes. They found mixed potential public health impact, with both the highest proportion of people likely to quit in a full flavoured tobacco and e-cigarette ban, but also the highest proportion of people who currently use flavoured products likely to switch to non-flavoured smoking. Wagener *et al* 15 examined potential combustible menthol alternatives in the case of a US menthol ban. A preassembled menthol roll-your-own cigarette with menthol pipe tobacco and mentholated cigarette tube was the most preferred product, highlighting the need to include these in the proposed FDA menthol ban. Aided by tobacco industry scare tactics, the spectre of increased illicit tobacco trade hangs over the introduction of most tobacco control policies. Chung-Hall *et al’s* study of illicit purchasing after Canada’s menthol bans rebuts this.^16^ Based on International Tobacco Control data, they found that there was no increase in purchasing of illicit menthol or other illicit cigarettes in Canada following the bans. Among those who did report post-ban use of menthol cigarettes (19.5%), brand analysis showed the true figure to be only 10.5%.

Four studies explore e-cigarettes. Pennings *et al* 17 propose a restrictive list of flavourings for e-cigarette liquids which would only enable a tobacco flavour to be produced, and prevent production of sweet or fruity flavours. Created to support a Dutch government decision to only allow tobacco flavours in e-liquids, several criteria were applied to exclude non-tobacco-related flavourings and those that were harmful for health. The resulting list included 16 flavours from the 506 that were notified to the Dutch government at the time—creating a tool that has the potential to set parameters for manufacturers and limit constant changes in e-cigarette products. Roberts *et al’s* 18 study of organised opposition by Juul to prevent local flavour bans shows the challenges of implementing such parameters, while Ng *et al* highlight the ubiquity of exposure to e-cigarette marketing and how it can create positive perceptions of products, even in markets where they are completely banned.^19^ Using waves 1 to 5 data from the Population and Health study, Krishnan *et al* 20 modelled trajectories of electronic nicotine delivery systems (ENDS) and cigarette use and found that prior to 2019, ENDS use did not contribute substantially to increased population-level smoking cessation. They note ongoing ENDS market changes as a factor to be considered in monitoring trajectories of dual use. Given the polarised perspectives on ENDS and their impact on smoking prevalence, this will continue to be a topic of intense research and debate.

Other nicotine and related products present regulatory conundrums. Duren *et al* bring together regulatory approaches for nicotine pouches across 67 countries,^21^ highlighting the challenge of products that do not easily fit existing product categories. Moving from a global to local approach, Kong *et al* describe the emergence of ‘tobacco-free blunts’ in the USA,^22^ with youth-appealing features used to smoke cannabis. As with nicotine pouches, these do not fit well with existing product categories but could be included in expanded authority of regulatory agencies. Finally, returning to the issue of public reactions to tobacco control policies, Graham-DeMello and Hoek explore how adults who smoke might respond to the (then) planned New Zealand policy to greatly reduce tobacco retail outlets.^23^ While participants expected the policy would support reduced smoking, they identified potential negative social outcomes. The study provides important insights for countries planning more intensive tobacco control measures.

Together, the papers in this issue illuminate the complexity of tobacco control policy and regulation, and point to ways for policy makers to proactively rather than reactively regulate the tobacco industry and its products. It is worth noting that the most comprehensive policy of all would be to work towards phasing out the sale of *all*, and certainly the most harmful, tobacco products.^24^
